# The Impact of Preprocedural Blood Pressure on Outcome After M‐TEER: The Paradox or Something Else?

**DOI:** 10.1002/clc.70062

**Published:** 2024-12-09

**Authors:** Marijana Tadic, Leonhard Schneider, Nicoleta Nita, Dominik Felbel, Michael Paukovitsch, Mathias Gröger, Mirjam Keßler, Wolfang Rottbauer

**Affiliations:** ^1^ Klinik für Innere Medizin II Universitätsklinikum Ulm Ulm Germany

**Keywords:** blood pressure, interventional mitral valve repair, mitral regurgitation

## Abstract

**Objective:**

The aim of this study was to investigate the influence of systolic blood pressure (SBP) values on admission on the outcome of mitral transcatheter edge‐to‐edge repair (M‐TEER).

**Methodology:**

We included all patients who underwent interventional MV repair in our institution between January 2010 and October 2020. All data are obtained from the MiTra ULM registry. Based on SBP values measured on admission, all patients were divided into four groups: < 120, 120−129, 130−139, and ≥ 140 mmHg.

**Results:**

Eight hundred and fifty‐eight patients were included in this study. There were no major differences in demographic and clinical characteristics between the four observed groups. The patients with SBP on admission ≥ 140 mmHg had the lowest prevalence of functional MR and the highest LVEF. Higher SBP at admission (HR 0.74, 95% CI: 0.63−0.87) and preprocedural LVEF values (HR 0.99, 95% CI: 0.97−0.99) were predictors of lower 1‐year mortality but did not impact 1‐year hospitalization rate or MACE in the whole study population. When patients were separated into two groups according to the mechanisms of MR (functional and structural), the results showed that higher SBP on admission and better preprocedural LVEF were associated with significantly lower 1‐year CV mortality in both groups of patients, with functional and structural MR. Higher SBP at admission was also associated with lower 1‐year CV mortality (HR 0.73, 95% CI: 0.55−0.96) in patients with preserved ejection fraction (LVEF > 50%), but not with 1‐year rehospitalization and MACE.

**Conclusion:**

Higher SBP on admission (> 140 mmHg) is an independent predictor of a better 1‐year outcome in patients treated with M‐TEER. The effect of higher SBP on outcome after M‐TEER should be further investigated.

## Graphical abstract

Kaplan−Meyer curves illustrate 1‐year survival after M‐TEER in patients with different blood pressure groups. The survival significantly declined with reduction of baseline SBP, but it was statistically significant between patients with the highest and lowest SBP (SBP≤ 140 vs. SBP < 120 mmHg), as well as between patients with lowest SBP and those with 130 < SBP < 139 mmHg.

## 1 Introduction

Mitral valve transcatheter edge‐to‐edge‐repair (M‐TEER) became a standard of care in the treatment of functional and structural mitral regurgitation (MR) in a large number of patients. The burden of clinical evidence about its safety and favorable clinical short‐ and long‐term outcomes has been continuously increasing [1, 2]. There is an ongoing debate about strict clinical, echocardiographic, and hemodynamic criteria for M‐TEER, as well as a choice between existing devices. It is evident that lack of the official guidelines in this field makes challenging the selection of eligible patients, as well as a choice of appropriate device and follow‐up of patients after M‐TEER. However, the importance of clinical parameters on the outcome in M‐TEER patients remains to be determined.

Our study group previously reported the significance of lung disorders [3], as well as a higher incidence of composite endpoint in patients with diabetes undergoing M‐TEER [4], whereas the role of body weight remains controversial [5]. The impact of systolic blood pressure (SBP) has not been investigated so far. The major clinical trials and real‐world data used SBP as one of many independent variables in the multivariable statistical analyses to adjust their data, but they were not focused on SBP influence on the outcome in M‐TEER patients. Considering the pathology heterogeneity among the patients undergoing M‐TEER, as well as their age and comorbidities, the evaluation of SBP impact is not an easy task. Structural and functional MR tend to be evaluated separately in large studies due to large differences in etiology, hemodynamics, and clinical characteristics. Hypertension is one of the most frequent comorbidities in patients undergoing M‐TEER, irrespective of MR etiology, and therefore its influence on the outcome might be very important. Considering the fact that pre‐interventional SBP may be influenced by medications, its predictive value is of great importance, regardless of hypertension status.

The aim of the present investigation was to evaluate the effect of SBP and arterial hypertension on 1‐year outcomes in patients with functional and structural MR who underwent M‐TEER.

## 2 Methodology

This observational retrospective study involved 858 patients who had an M‐TEER procedure in the period from January 2010 to October 2020 at our institution. All involved participants had symptomatic moderate to severe and severe MR that was identified and evaluated by both, transthoracic and transesophageal echocardiography. Patients received optimized guideline‐directed medical therapy for heart failure and arterial hypertension at least 6 months before the M‐TEER procedure. All patients were assessed for the surgical risk and only those with high risk (EuroSCORE > 8%), who were not eligible for mitral valve surgery, were involved in this study. The multidisciplinary heart team was responsible for making final recommendation for transcatheter MV edge‐to‐edge repair according the current guidelines about valvular heart disease [6]. Preprocedural transoesophageal and transthoracic echocardiographic examination and left‐ and right‐heart catheterization including hemodynamic measurements were conducted, as the part of protocol for this type of procedure. However, cardiac catheterization was not the part of protocol during the whole study period, as it was not recommendation in the first years of experience with M‐TEER, and therefore complete invasive hemodynamic data were retrieved for 316 patients. Considering the cause of MR, all subjects were divided into two groups: primary (structural) MR and secondary (functional) MR [7].

The European System for Cardiac Operative Risk Evaluation (EuroSCORE II) was used for evaluation the risk for surgical repair of the MV [8]. The M‐TEER procedure was conducted in a hybrid catheterization laboratory under general anesthesia. M‐TEER was performed using MitraClip and PASCAL devices that were implanted using fluoroscopic and echocardiographic guiding.

The electronic medical records were used for retrieving demographic, clinical, and laboratory data, together with information regarding comorbidities and medications. All diseases were coded according to ICD‐9 and 10 classifications of disease. Patients were divided into four groups according to their baseline SBP values measured at the admission: < 120 mmHg (group I), 120−129 mmHg (group II), 130−139 mmHg (group III), and ≥ 140 mmHg (IV). SBP was measured by a digital sphygmomanometer (Riestar RBP‐100, Jungingen, Germany) on admission, in the morning hours, by averaging the two consecutive measurements in the sitting position, taken within an interval of 5 min, after the subject had rested for at least 5 min in that position.

Included patients are participants of the prospective MiTra ULM registry and they provided written informed consent for taking the part in this study. The local ethical committee provided approval for the research protocol.

### 2.1 Patients' Follow‐Up

Standardized patients' follow‐up was completed during routine clinical visits at 1, 3, 6, and 12 months. Patients were routinely evaluated at the outpatient department.

### 2.2 Statistical Analysis

Continuous variables were presented as mean ± standard deviation. Continuous variables between four different groups were compared by the analysis of equal variance (ANOVA), as they showed normal distribution using the Kolmogorov–Smirnov test. Tukey post hoc analysis was used for the comparison between different groups. Differences in proportions were compared by the *χ*² test or Fischer's exact test where appropriate. Kaplan–Maier curves and the log‐rank test were used to analyze the impact of different variables on endpoints (cardiovascular mortality, hospitalization due to heart failure, and MACE). MACE is considered as a composite endpoint that consists of cardiovascular mortality, myocardial infarction, and stroke. Univariate and multivariate Cox proportional hazards regression was used to quantify the impact of these parameters on outcomes. The two‐tail *p* < 0.05 was considered statistically significant.

## 3 Results

There was no significant difference in majority of demographic and clinical characteristics between patients with different SBP level (Table 1). Males were the least prevalent in group with the lowest SBP on admission. Parameters of renal function (creatinine, GFR) were the lowest among patients with baseline SBP < 120 mmHg and prevalence of renal failure was the highest in this group of patients (Table 1). LV diameters were the smallest and LVEF was the highest among patients with SBP > 140 mmHg.

**Table 1:** Demographic characteristics and clinical parameters of study population.

	Group I (SPB < 120 mmHg) (*n* = 293)	Group II (SBP 120−130 mmHg) (*n* = 163)	Group III (SBP 130−140 mmHg) (*n* = 168)	Group IV (SBP > 140 mmHg) (*n* = 234)	*p*
Age (years)	75 ± 10	77 ± 7^a^	78 ± 7^a^	79 ± 8^a^	< 0.001
Male (%)	105 (36)	67 (41)	71 (42)	112 (48)^b^	0.049
BMI (kg/m^2^)	25.9 ± 4.7	25.4 ± 4.5	25.6 ± 5.2	26.1 ± 4.4	0.448
Obesity (%)	47 (16)	23 (14)	31 (18)	44 (19)	0.570
Heart rate (beats/min)	75 ± 16	72 ± 16	74 ± 16	73 ± 14	0.130
SBP on admission (mmHg)	104 ± 11	124 ± 2	134 ± 2	150 ± 9	< 0.001*
DBP on admission (mmHg)	65 ± 9	71 ± 9	75 ± 8	81 ± 11	< 0.001*
NYHA class					
III (%)	204 (70)	120 (74)	125 (74)	180 (77)	0.255
IV (%)	89 (30)	44 (26)	43 (26)	54 (23)	
Interventions and surgeries					
PCI (%)	128 (44)	70 (43)	81 (48)	114 (49)	0.500
CABG (%)	48 (16)	31 (19)	28 (17)	36 (15)	0.740
Mitral valve surgery (%)	3 (1)	1 (0.6)	0 (0)	2 (1)	0.630
TAVR (%)	13 (4)	11 (7)	14 (8)	17 (7)	0.355
Aortic valve surgery (%)	12 (4)	9 (6)	10 (6)	17 (7)	0.583
Comorbidities					
CAD (%)	189 (65)	109 (67)	120 (71)	156 (67)	0.437
Previous MI (%)	71 (24)	38 (23)	51 (30)	45 (19)	0.170
Hypertension (%)	213 (73)	133 (82)^b^	148 (88)^a^	200 (85)^a^	< 0.001
Dyslipidemia (%)	170 (58)	90 (55)	111 (66)	139 (59)	0.193
Diabetes (%)	82 (28)	59 (36)	50 (30)	55 (24)	0.057
Smoking (%)	30 (10)	9 (6)	9 (5)	15 (6)	0.097
Atrial fibrillation (%)	188 (64)	90 (55)	117 (70)	143 (61)	0.167
Peripheral artery disease (%)	23 (8)	18 (11)	16 (10)	19 (8)	0.675
COPD (%)	44 (15)	16 (10)	22 (13)	20 (9)	0.103
OSAS (%)	24 (8)	8 (5)	8 (5)	13 (6)	0.367
Peptic ulcer disease (%)	6 (2)	6 (4)	3 (2)	4 (2)	0.565
Renal failure (%)	164 (56)	83 (51)	92 (55)	99 (42)^a^,^c^	0.012
Acute renal failure (%)	17 (6)	5 (3)	5 (3)	13 (6)	0.341
Hepatic cirrhosis (%)	4 (1)	1 (0.6)	2 (1)	4 (2)	0.813
Previous cancer (%)	62 (21)	27 (17)	22 (13)	35 (15)	0.196
Antiarrhythmia devices					
CRT (%)	41 (14)	16 (10)	12 (7)	10 (4)^a^,^d^	0.001
ICD (%)	64 (22)	18 (11)^a^	19 (11)^a^	16 (7)^a^	< 0.001
Pacemaker (%)	27 (9)	7 (4)	18 (11)	26 (11)	0.097
Scores					
Euro score II	9.5 ± 9.4	8.1 ± 6.8	8.0 ± 7.4^a^	7.2 ± 7.7^a^	0.013
Therapy					
ACEI (%)	124 (42)	76 (47)	78 (46)	108 (46)	0.767
ARB (%)	78 (27)	45 (71)	42 (25)	75 (32)	0.404
ARNI (%)	24 (8)	15 (9)	12 (7)	6 (3)^a^,^e^	0.010
Beta‐blockers (%)	254 (87)	147 (90)	150 (89)	187 (80)^b^,^c^,^e^	0.013
Aldosterone antagonists (%)	151 (51)	89 (55)	73 (43)	74 (32)^a^,^c^,^e^	< 0.001
Statins (%)	188 (64)	107 (66)	111 (66)	164 (70)	0.559
Laboratory					
Creatinine (μmol/L)	143 ± 78	135 ± 82	126 ± 60^b^	116 ± 53^a^,^b^	< 0.001
GFR (mL/min/1.73 m^2^)	46 ± 20	47 ± 19	49 ± 18	52 ± 19^a^	0.004
NT‐pro‐BNP (pg/mL)	6226 ± 7677	4831 ± 4910^a^	5535 ± 6075	5281 ± 6354^d^	0.014
Troponin (ng/L)	126 ± 911	41 ± 42	52 ± 153	53 ± 265	0.376
Echocardiography					
Functional MR (%)	179 (61)	110 (67)	111 (66)	127 (54)^c^,^d^	0.032
LVEF (%)	38 ± 15	43 ± 14	45 ± 15^a^	51 ± 16^a^,^d^,^f^	< 0.001
LVEDD (mm)	62 ± 12	61 ± 10	61 ± 11^a^	56 ± 10^a^,^d^,^f^	< 0.001
LVESD (mm)	45 ± 15	46 ± 12	43 ± 12^a^,^e^	45 ± 14	< 0.001
Interventricular septum thickness (mm)	11 ± 2	11 ± 2	11 ± 4	12 ± 4	0.114
LA (mm)	56 ± 9	56 ± 8	55 ± 10	54 ± 9	0.242
Intraprocedural results					
MR severity after TEER					
MR ≤ 1+	248 (85)	140 (86)	148 (88)	206 (88)	0.625
MR 2+	18 (6)	13 (8)	11 (7)	11 (5)	0.608
MR 3+	27 (9)	10 (6)	9 (5)	17 (7)	0.418
Mitral stenosis (%)	6 (2)	2 (1)	2 (1)	3 (1)	0.558

Abbreviations: ACEI, angiotensin‐converting enzyme inhibitor; ARB, angiotensin II receptor blocker; ARNI, angiotensin receptor II blocker‐neprilysin inhibitor; BMI, body mass index; CABG, coronary artery bypass grafting; COPD, chronic obstructive pulmonary disease; CRT, cardiac resynchronization therapy; GFR, glomerular filtration rate; ICD, implantable cardiac defibrillators; LA, left atrium; LV, left ventricle; LVEDD, left ventricular end‐diastolic diameter; LVEF, left ventricular ejection fraction; LVESD, left ventricular end‐systolic diameter; MI, myocardial infarction; MR, mitral regurgitation; OSAS, obstructive sleep‐apnea syndrome; PCI, percutaneous coronary artery intervention; TAVR, transcatheter aortic valve replacement; TEER, transcatheter edge‐to‐edge repair.

* *p* < 0.01 for comparisons between all groups.

^a^ *p* < 0.01 versus group I.

^b^ *p* < 0.05 versus group I.

^c^ *p* < 0.05 versus group III.

^d^ *p* < 0.05 versus group II.

^e^ *p* < 0.01 versus group II.

^f^ *p* < 0.01 versus group III.

The majority of hemodynamic parameters showed no difference between four observed groups (Table 2). The only differences were seen in systemic resistance and systolic BP which were significantly higher in patients with SBP > 140 mmHg.

**Table 2:** Hemodynamic measurements in study population.

	Group I (SPB < 120 mmHg) (*n* = 115)	Group II (SBP 120−130 mmHg) (*n* = 53)	Group III (SBP 130−140 mmHg) (*n* = 62)	Group IV (SBP > 140 mmHg) (*n* = 86)	*p*
Mean RA pressure (mmHg)	11 ± 7	11 ± 8	12 ± 6	10 ± 6	0.682
Mean RV pressure (mmHg)	26 ± 11	22 ± 13	32 ± 18	24 ± 14	0.202
Systolic PA pressure (mmHg)	50 ± 15	49 ± 15	53 ± 17	50 ± 17	0.539
Diastolic PA pressure (mmHg)	19 ± 8	20 ± 13	20 ± 9	19 ± 11	0.832
Mean PA pressure (mmHg)	33 ± 9	32 ± 10	34 ± 11	34 ± 21	0.719
Mean PCWP (mmHg)	24 ± 9	22 ± 8	24 ± 10	21 ± 9	0.152
Mean LA pressure (mmHg)	19 ± 9	18 ± 7	23 ± 10	18 ± 8	0.064
LV end‐systolic pressure (mmHg)	130 ± 28	118 ± 29	137 ± 23	142 ± 38	0.577
LV end‐diastolic pressure (mmHg)	19 ± 7	21 ± 10	20 ± 7	19 ± 7	0.364
SBP on admission (mmHg)	114 ± 30	119 ± 21	133 ± 24^a^,^c^	140 ± 30^a^,^c^	< 0.001
DBP before procedure (mmHg)	62 ± 15	64 ± 14	67 ± 12	76 ± 15	0.211
Mean BP (mmHg)	83 ± 20	85 ± 15	89 ± 20^a^	93 ± 22^a^,^c^	< 0.001
SVR (dynes/s/cm^−5^)	1784 ± 870	1698 ± 614	2034 ± 800^a^, ^c^	2665 ± 726^a^, ^c^, ^d^	< 0.001
PVR (dynes/s/cm^−5^)	312 ± 243	299 ± 210	321 ± 303	325 ± 259	0.201
Cardiac output (L/min)	3.8 ± 1.2	3.8 ± 0.9	4.0 ± 1.2	3.8 ± 1.2	0.841
Cardiac index (L/min/m^2^)	2.0 ± 0.6	2.1 ± 0.4	2.2 ± 0.6	2.1 ± 0.6	0.550
Oxygen saturation in aorta (%)	91 ± 4	92 ± 4	89 ± 5^a^,^c^	90 ± 4^e^	< 0.001
Oxygen saturation in PA (%)	56 ± 9	59 ± 9	56 ± 9	58 ± 9	0.088

Abbreviations: BP, blood pressure; LA, left atrial; LV, left ventricular; MR, mitral regurgitation; PA, pulmonary artery; PCWP, pulmonary capillary wedge pressure; PVR, pulmonary vascular resistance; SVR, systemic vascular resistance.

^a^ *p* < 0.01 versus group I.

^b^ *p* < 0.05 versus group II.

^c^ *p* < 0.01 versus group II.

^d^ *p* < 0.01 versus group III.

^e^ *p* < 0.05 versus group I.

Patients with higher SBP on admission were at lower risk of 1‐year CV mortality, but not for hospitalization rate and MACE (Table 3). The results remain the same in multivariable analysis and only higher SBP on admission and preprocedural LVEF were predictors of lower mortality risk, while age, gender, diabetes, obesity, use of beta‐blockers and ARNI, as well as good M‐TEER results defined by post‐interventional MR1+, were not predictors of poor outcome. In group of patients with preserved ejection fraction (LVEF > 50%), higher SBP levels were also related with lower CV mortality (HR 0.73, 95% CI: 0.55−0.96, *p* = 0.026), but they were not predictors of 1‐year rehospitalization and MACE.

**Table 3:** Clinical outcomes during 1‐year follow‐up.

	CV mortality		Hospitalization rate			MACE
	HR	95% CI	HR	95% CI	HR	95% CI
Age (years)	1.02	0.99−1.04	1.00	0.99−1.01	0.99	0.98−1.01
Gender (men)	0.78	0.54−1.15	0.98	0.77−1.25	1.29	1.04−1.59
SBP on admission (groups)	0.74	0.63−0.87	0.98	0.89−1.08	0.94	0.73−1.21
Hypertension	0.79	0.35−1.76	0.77	0.59−1.98	1.05	0.82−1.82
Diabetes	1.13	0.77−1.67	0.99	0.78−1.27	0.87	0.70−1.08
Obesity	1.16	0.73−1.84	0.98	0.72−1.33	1.06	0.83−1.37
CRT	0.86	0.74−1.56	0.92	0.75−1.40	0.83	0.69−1.10
Beta‐blockers	0.78	0.48−1.26	0.99	0.70−1.41	1.04	0.78−1.39
ARNI	1.08	0.52−2.26	0.95	0.62−1.45	1.24	0.83−1.86
LVEF (%)	0.98	0.97−0.99	1.00	0.99−1.01	1.00	0.99−1.01
MR ≤ 1+	0.96	0.64−1.44	0.97	0.75−1.27	1.05	0.84−1.32

Abbreviations: ARNI, angiotensin receptor II blocker‐neprilysin inhibitor; LVEF, left ventricular ejection fraction; MR, mitral regurgitation; OSAS, obstructive sleep‐apnea syndrome; PCI, percutaneous coronary artery intervention; TAVR, transcatheter aortic valve replacement.

Slightly different results were obtained when all patients were divided into two groups: functional and structural MR (Table 4). However, only higher SBP was the independent predictor of lower 1‐year CV mortality (HR: 0.86, 95%: 0.71−0.97, *p* = 0.029) in a multivariate analysis that included age, gender, diabetes, obesity, type of MR (functional vs. structural), use of beta‐blockers, ACEI/ARB, ARNI, aldosterone antagonists, GFR, NT‐pro‐BNP, LVEDD, LVEF, MR severity, EuroSCORE II, and systemic vascular resistance. Interestingly, the only independent predictor of MACE was sex (male).

**Table 4:** Clinical outcomes during 1‐year follow‐up in patients with functional MR.

	Functional MR						Structural MR					
	CV mortality		Hospitalization rate		MACE		CV mortality		Hospitalization rate		MACE	
	HR	95% CI	HR	95% CI	HR	95% CI	HR	95% CI	HR	95% CI	HR	95% CI
Age (years)	1.01	0.98−1.04	0.99	0.98−1.01	0.99	0.98−1.00	1.03	0.99−1.08	1.01	0.99−1.04	1.01	0.99−1.03
Gender (men)	0.58	0.35−0.98	1.34	0.96−1.85	1.22	0.93−1.60	1.14	0.65−2.02	1.32	0.88−1.99	1.41	1.01−1.98
SBP on admission (groups)	0.73	0.59−0.91	0.99	0.88−1.14	0.97	0.86−1.09	0.75	0.59−0.95	0.98	0.83−1.15	0.99	0.87−1.14
Hypertension	1.18	0.75−1.94	0.95	0.71−1.74	1.05	0.87−1.83	1.08	0.62−1.85	0.84	0.47−2.15	1.16	0.92−1.90
Diabetes	1.12	0.69−1.13	1.01	0.73−1.40	0.95	0.72−1.24	1.06	0.80−1.41	0.81	0.68−0.98	0.88	0.75−1.03
Obesity	1.58	0.90−2.76	1.17	0.79−1.74	1.22	0.88−1.69	0.68	0.29−1.59	0.76	0.48−1.21	0.87	0.58−1.30
CRT	0.92	0.79−1.30	0.86	0.65−1.25	0.89	0.70−1.15	0.82	0.69−1.22	0.83	0.63−1.33	0.95	0.77−1.23
Beta‐blockers	0.53	0.30−0.91	0.52	0.31−0.86	0.75	0.52−1.10	1.50	0.64−3.52	1.36	0.80−2.32	1.40	0.89−2.21
ARNI	1.59	0.74−3.44	1.53	0.88−2.65	1.33	0.81−2.18	0.85	0.49−2.31	1.16	0.56−2.42	1.04	0.50−2.14
LVEF (%)	0.98	0.96−0.99	0.99	0.99−1.01	1.00	0.99−1.01	0.98	0.97−0.99	1.01	0.99−1.02	1.01	0.99−1.02
MR≤ 1+	1.20	0.75−2.01	0.97	0.68−1.41	1.16	0.87−1.54	0.64	0.31−1.32	0.75	0.48−1.16	0.88	0.61−1.29

Abbreviations: ARNI, angiotensin receptor II blocker‐neprilysin inhibitor; LVEF, left ventricular ejection fraction; MR, mitral regurgitation; OSAS, obstructive sleep‐apnea syndrome; PCI, percutaneous coronary artery intervention; TAVR, transcatheter aortic valve replacement.

**Figure 1:**
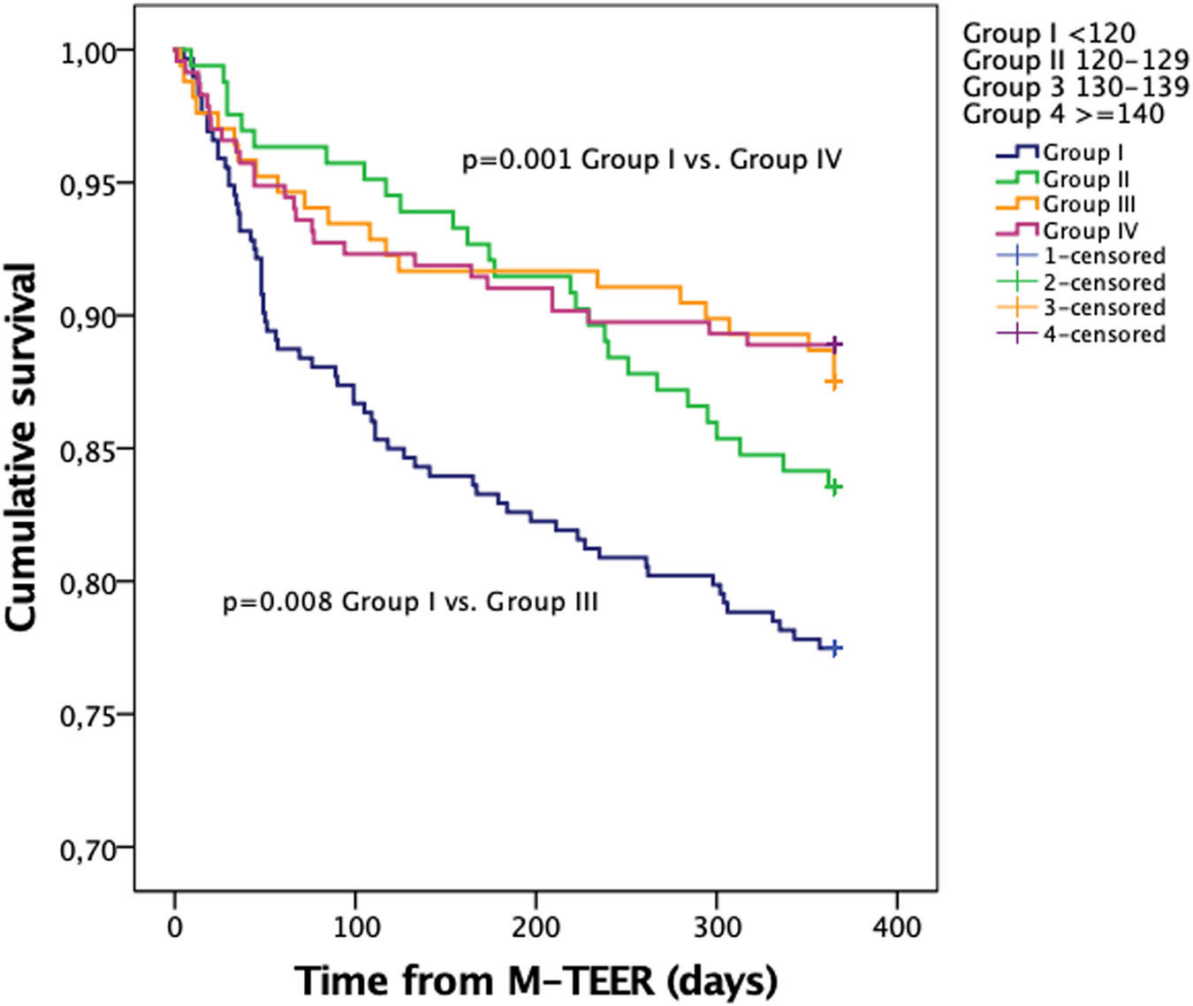
One‐year survival after M‐TEER in patients with different blood pressure groups.

Figure 1 illustrates Kaplan−Meyer curve and survival among four different groups of patients with various SBP on admission. The survival significantly declined with reduction of baseline SBP, but it was statistically significant between patients with the highest and lowest SBP (SBP ≤ 140 vs. SBP < 120 mmHg), as well between patients with lowest SBP and those with 130 < SBP < 139 mmHg. The same analysis showed no difference in 1‐year survival between patients with functional and structural MR (*p* = 0.928). A significant difference was not found in 1‐year survival between patients with LVEF > 50% and LVEF < 50% (*p* = 0.201).

## 4 Discussion

The present study revealed several important findings that will be further discussed: (i) there was no significant differences in majority of demographic, clinical, and hemodynamic characteristics between groups with different SBP on admission; (ii) baseline SBP is a significant predictor of 1‐year CV mortality in patients undergoing M‐TEER; (iii) patients with SBP on admission < 120 mmHg had significantly higher 1‐year cardiovascular mortality risk than patients with SBP 130−139 mmHg and SBP≥ 140 mmHg, even after adjustment for preprocedural LVEF; (iv) baseline SBP < 120 mmHg, along with preprocedural LVEF, was the independent predictor of CV mortality, but not predictor of hospitalization due to heart failure or MACE, in the whole group of patients who underwent M‐TEER, as well as in patients with functional and structural MR, separately.

The influence of SBP on the outcome of M‐TEER has not been sufficiently investigated. The majority of patients who underwent this procedure have many comorbidities, but all studies and trials confirm that hypertension represents the most frequent (70%−85%) [1, 2, 3, 4, 5, 9, 10, 11]. This high prevalence of hypertension was also detected in the present study. Previous studies included hypertension in a multivariate analysis to adjust their result, but the effect of SBP itself was not assessed. The number of comorbidities, particularly heart and renal failure, as well as hemodynamic changes in MR patients, particularly before, during, and immediately after surgery are significant confounding factors for evaluation of isolated BP impact on outcome in these patients. This was the reason for performing several sub‐analyses taking into account the MR type (structural vs. functional) and LVEF (normal vs. reduced).

To determine the impact of SBP on the outcome in M‐TEER, it is necessary to understand hemodynamic changes caused by M‐TEER. Namely, previously was believed that M‐TEER improves general hemodynamics by causing significant LV unloading [12, 13, 14]. This is reflected by immediate decrease in end‐diastolic pressure and increase in end‐systolic wall stress [12, 13, 14]. This was followed by reduction in pulmonary capillary wedge pressure and elevation in cardiac index [12, 13, 14]. Acute reduction in LV preload and concomitant increase in ventricular afterload might be responsible for an increase in potential energy which is stored in the myocardium and consequently decreased efficiency of the myocardium to transfer energy to myocardial work. This so‐called *afterload mismatch* is believed to be responsible for LVEF decline in some M‐TEER patients. The authors from the MITRA‐EF study defined *afterload mismatch* as a relative LVEF reduction of 15% as the cut‐off for significant LVEF decline after M‐TEER and reported this finding in 18.5% of all patients who underwent M‐TEER [15]. They reported significantly higher mortality, hospitalization, and MACE rates in patients with *afterload mismatch* only in patients with structural MR, but not in those with functional MR or in the whole cohort of patients [15]. The authors also found that systolic SBP > 120 mmHg and higher LVEF were independent predictors of acute LVEF reduction in patients with structural MR [15]. Considering the fact that LVEF was significantly higher among patients with structural MR, one would expect that acute LVEF decline would impact more patients with functional MR with already reduced LVEF.

On the other hand, our results showed that patients with higher baseline SBP had better outcomes than those with SBP < 120 mmHg. Namely, patients with higher BP have higher invasively‐measured systemic vascular resistance and therefore higher afterload, which theoretically makes them more susceptible to *afterload mismatch* and potential LVEF decline that is related with higher mortality. However, our results reported better outcomes in patients with higher baseline SBP in the whole study population, as well as in both groups—structural and functional MR patients. There are several potential reasons for this result. First, the authors of the MITRA‐EF study did not use SBP values but only used a cut‐off of 120 mmHg in statistical analysis. Our study showed that outcomes of M‐TEER patients start to differentiate with baseline SBP > 130 mmHg. There was no difference between patients with SBP < 120 mmHg and those with SBP between 120 and 129 mmHg. Second, the information about the prevalence of LVEF reduction after M‐TEER is not available for our population. We can speculate that *afterload mismatch* prevalence is significantly lower in our study and therefore does not affect the outcome in our cohort of patients. Third, the large percentage of our patients have heart failure, particularly those with functional MR, and previous meta‐analysis showed that 10 mmHg higher SBP was related with 13.0% reduction (95% CI: 10.6%−15.4%) in mortality rate in patients with heart failure [16]. This might partly explain the effect of higher SBP in M‐TEER patients.

The increase in SBP is likely to represent an improvement in cardiac output. Considering the fact that SBP in patients with FMR and heart failure, unlike in the general population, is more significantly related with cardiac output than peripheral resistance, this increase in systolic SBP is likely to reflect an improved cardiac output and ejection fraction. However, it is questionable if higher SBP is a reasonable aim for long‐term outcomes because of the general perception in cardiology that lower blood pressure is better and associated with less target organ damage. The clinical implication of this study remains to be determined in large trials with long follow‐ups. Our preliminary results suggest that we should be careful with SBP control and not suggest strict regulation with SBP < 120 mmHg, as this may be harmful for MR patients who are planned for M‐TEER. Larger studies with longer outcomes and preferably trials would provide more details on this topic.

### 4.1 Limitations

The current investigation is related with some limitations that should be mentioned. All data were obtained from the local registry and not randomized clinical trial, which is related with certain limitations and biases typical for a single‐center study. However, this is a real population of patients with common comorbidities and therefore the inclusion of all patients represents an important strength of this investigation. Our study did not investigate the influence of races, as the vast majority of our patients were white, which might be considered as additional limitation. Mechanisms that are responsible for predictive value of SBP on the outcome of M‐TEER are unknown and it should be cautious with generalization of these results before large prospective trials confirm our preliminary data.

## 5 Conclusion

The present study showed that higher SBP on admission and preprocedural LVEF were predictors of better 1‐year survival, but did not impact 1‐year hospitalization rate or MACE composite outcome in all patients who underwent M‐TEER. SBP on admission remained an independent predictor even when patients were divided into two groups according to MR types (functional vs. degenerative) or preprocedural LVEF (< 50% vs. > 50%). Therefore, results indicate that strict SBP control and reduction of SBP below 120 mmHg before M‐TEER might be reconsidered in the future. The SBP on admission represents an important predictor of outcome in different types of M‐TEER patients and should be included in preprocedural evaluation and prediction of long‐term outcomes, not only as part of the calculated score but also individually. Further longitudinal studies and trials are necessary to establish the relationship between baseline SBP and short‐ and long‐term outcomes in M‐TEER patients.

## Conflicts of Interest

The authors declare no conflicts of interest.

## Data Availability

The data that support the findings of this study are available on request from the corresponding author. The data are not publicly available due to privacy or ethical restrictions.
